# Local structural-functional connectivity decoupling of caudate nucleus in infantile esotropia

**DOI:** 10.3389/fnins.2022.1098735

**Published:** 2022-12-22

**Authors:** Jianlin Guo, Yuanyuan Chen, Lijuan Huang, Wen Liu, Di Hu, Yanqiu Lv, Huiying Kang, Ningdong Li, Yun Peng

**Affiliations:** ^1^Imaging Center, MOE Key Laboratory of Major Diseases in Children, Beijing Children’s Hospital, National Center for Children’s Health, Capital Medical University, Beijing, China; ^2^Tianjin International Joint Research Center for Neural Engineering, Academy of Medical Engineering and Translational Medicine, Tianjin University, Tianjin, China; ^3^Department of Ophthalmology, Beijing Children’s Hospital, National Center for Children’s Health, Capital Medical University, Beijing, China; ^4^Department of Ophthalmology, The Second Affiliated Hospital of Fujian Medical University, Quanzhou, China; ^5^Key Laboratory of Major Diseases in Children, Ministry of Education, Beijing, China

**Keywords:** infantile esotropia, functional connectivity, structural connectivity, coupling, caudate nucleus

## Abstract

Abnormal brain structural and functional properties were demonstrated in patients with infantile esotropia (IE). However, few studies have investigated the interaction between structural and functional connectivity (SC-FC) in patients with IE. Structural network was generated with diffusion tensor imaging and functional network was constructed with resting-state functional magnetic resonance imaging for 18 patients with IE as well as 20 age- and gender- matched healthy subjects. The SC-FC coupling for global connectome, short connectome and long connectome were examined in IE patients and compared with those of healthy subjects. A linear mixed effects model was employed to examine the group-age interaction in terms of the coupling metrics. The Pearson correlation between coupling measures and strabismus degree was evaluated in IE patients, on which the regulatory effect of age was also investigated through hierarchical regression analysis. Significantly decreased SC-FC coupling score for short connections was observed in left caudate nucleus (CAU) in IE patients, whereas no brain regions exhibited altered coupling metrics for global connections or long connections. The group-age interaction was also evident in local coupling metrics of left CAU. The age-related regulatory effect on coupling-degree association was distinguishing between brain regions implicated in visual processing and cognition-related brain areas in IE patients. Local SC-FC decoupling in CAU was evident in patients with IE and was initiated in their early postnatal period, possibly interfering the visual cortico-striatal loop and subcortical optokinetic pathway subserving visual processing and nasalward optokinesis during neurodevelopment, which provides new insight into underlying neuropathological mechanism of IE.

## Introduction

Infantile esotropia (IE) is a subtype of concomitant strabismus manifesting within the first 6 months of life and is characterized by constant and large-angle esodeviation, alternate fixation, impaired stereovision and dissociated eye movements (e.g., dissociated vertical deviation and latent nystagmus), with no evidence of additional neurological disease (Brodsky, 2018). As a rare condition with 2/1,000 prevalence, IE not only interferes the normal neurodevelopment of the vision but also influences the cosmetic appearance of the children (Torp-Pedersen et al., 2017). The specific pathogenesis of IE remains poorly understood. Further understanding the neuropathological mechanism of IE is conducive for treatment option and prognosis evaluation.

Previous animal studies have revealed structural and functional alterations in visual areas of brain in various species of animals with experimentally induced strabismus (Kiorpes et al., 1998; Zhang et al., 2005; Schmidt and Löwel, 2008). In recent years, human neuroimaging studies have explored the alterations of brain structure in patients with strabismus. For instance, it was reported that patients with strabismic amblyopia exhibited tract-specific changes in mean diffusivity (MD), especially the optic radiation, vertical occipital fasciculus, frontal corpus callosum and some long-range association tracts (Duan et al., 2015). Decreased MD along the optic radiation was also observed in patients with constant exotropia (Wang et al., 2022). In addition to the structural alterations, strabismic patients also exhibited functional changes of the brain, which were demonstrated by resting-state functional MRI (rsfMRI) studies. For example, reduced functional network connectivity of the primary visual cortex with numerous brain regions related to vision processing and eye movement was found in patients with intermittent exotropia (He et al., 2021). Through an independent component analysis, altered intra- and inter- network connectivity were revealed in patients with concomitant exotropia, in terms of the visual network, sensorimotor network and auditory network (Jin et al., 2022).

However, no studies have explored the coupling metrics of brain structural and functional profiles in strabismus. As an indicator implicated in the combined analysis of structural connectivity (SC) and functional connectivity (FC) networks, SC-FC coupling is intrinsically a reflection of the structural-functional coherence, which has shown potential in quantifying the changes of structural-functional network association across age during development (Hagmann et al., 2010) and has been applied to investigate a variety of neurological disorders in recent years (Sun et al., 2017; Zhang et al., 2019; Chen et al., 2021). Evidence suggests that SC-FC coupling serves as a reliable and valuable index surpassing any single-modal network property (Zhang et al., 2011). Distinct alterations of coupling metrics were demonstrated in various conditions. For instance, alterations of SC-FC coupling strength in terms of the long connections were demonstrated in children with a family history of schizophrenia and bipolar disorder (Collin et al., 2017). In addition, abnormality in SC-FC coupling for short connections was reported in cannabis users (Kim et al., 2019). Furthermore, patients with idiopathic tinnitus exhibited altered SC-FC coupling metrics in global network connections (Chen et al., 2022). Nonetheless, it is still unclear if the patients with IE may present altered SC-FC coupling metrics for global connections, short connections or long connections. To fill in this gap and further illustrating the role of coupling metrics in the neurobiological process of IE, this study investigated the SC-FC coupling profiles for each of the three connectomes across whole brain subregions.

In the current study, our coupling analysis mainly included two steps: we first compared the SC-FC coupling in IE patients with that of healthy subjects, of which the group-age interaction was also examined to identify distinct developmental trends of coupling metrics in IE. Afterward, we assessed the correlation of the coupling measures with clinical variable, on which the age-related regulatory effect was also investigated in different brain subregions. Abnormalities of structural neuroimaging of strabismic patients were overlapped for regions implicated in visual pathway (Duan et al., 2015; Wang et al., 2022). Thus, we hypothesized that patients with IE would show unique pattern of SC-FC coupling in brain, especially in those local regions involved in visual information processing, and exhibit distinct developmental trajectory of coupling profile across age.

## Materials and methods

### Participants

A total of 18 patients with IE (10 males and 8 females, mean age: 3.27 ± 2.00, age range: 0.41–6.36) were enrolled in this study from Beijing Children’s Hospital. Meanwhile, 20 age- and gender- matched healthy subjects (11 males and 9 females, mean age: 3.41 ± 1.58, age range: 1.18–6.28) were also recruited as controls. The inclusion criteria for IE were as follows: (1) large-angle nasalward deviation of both eyes manifesting before 6 months of age; (2) free of other eye diseases or previous ophthalmic surgery; (3) without neuropsychiatric or cardiovascular disease; (4) full-term birth. Subjects who met one of the following conditions were excluded: (1) brain abnormality detected by conventional MRI; (2) contraindications for MR examination. The research conformed to the Declaration of Helsinki and was approved by the Medical Research Ethic Committee of Beijing Children’s Hospital. Written informed consents for each participate were obtained from their parents before any procedures of the study.

### MRI data acquisition

Imaging data were collected using a 3.0 Tesla MRI system (Discovery MR 750, General Electric, Milwaukee, WI, USA) in Medical Imaging Center of Beijing Children’s Hospital. All participates underwent scanning during natural sleep. Earplugs and spongy cushions were used to cut down on the noise and minimize head motion during scanning. A camera facing to the scanner bed was applied to monitor the sleeping behaviors of the subjects. T1-weighted, sagittal three-dimension brain volume sequence was acquired with following parameters: repetition time (TR) = 8.2 ms, echo time (TE) = 3.2 ms, flip angle = 12°, field of view (FOV) = 256 mm × 256 mm, acquisition matrix = 256 × 256, slice thickness = 1 mm, voxel size = 1 mm × 1 mm × 1 mm, slice = 164. The rsfMRI data were collected with echo-planar imaging (EPI) sequence, and the scanning parameters were as follows: TR = 2,000 ms, TE = 30 ms, FOV = 224 mm × 224 mm, acquisition matrix = 64 × 64, slice thickness = 3.5 mm, voxel size = 3.5 mm × 3.5 mm × 3.5 mm, flip angle = 90°, slice = 40, data volume = 240. Axial diffusion tensor imaging (DTI) was acquired with a single-shot EPI sequence: TR = 7,200 ms, TE = 63 ms, slice thickness = 2 mm, slice gap = 0 mm, slice = 72, acquisition matrix = 128 × 128, FOV = 256 mm × 256 mm, voxel size = 2 mm × 2 mm × 2 mm, 32 diffusion gradient direction images with the *b*-value of 1,000 s/mm^2^ and a single image with the *b*-value of 0 s/mm^2^.

### DTI data preprocessing

Diffusion tensor imaging brain imaging data were preprocessed by FMRIB Software Library (FSL 6.0)^1^ (Jenkinson et al., 2012) and FreeSurfer package^2^ (Fischl, 2012). The main steps were as follows: (1) skull-stripping to remove the non-brain tissue with FreeSurfer package, which is better and more stable than FSL-bet; (2) eddy current correction and motion correction with FSL-eddycorrect. Framewise displacement (FD) was used to assess the head motion, and datasets with mean FD bigger than 0.3 mm were excluded in the following analysis (Power et al., 2014); (3) tensor estimation was performed with FSL-dtifit, including fractional anisotropy (FA), mean diffusivity (MD), axial diffusivity (AD), and radial diffusivity (RD).

### rsfMRI data preprocessing

Analysis of Functional NeuroImages (AFNI)^3^ was used in rsfMRI data preprocessing. The main steps of preprocessing pipeline included: (1) removing the first 5 time points; (2) slice timing correction to remove the time difference between slices; (3) head motion correction with linear registration. Scrubbing method was applied removing the volumes with FD >0.3 mm and less than 3 continuous timespoints; (4) nuisance covariates regression of white matter signal, cerebral fluid signal, global signal and Friston-24 head motion parameters (Friston et al., 1996); (5) band-pass filtering with the frequency range of 0.01–0.1 Hz; (6) spatial smoothing with a 6 mm full width at a half maximization Gaussian kernel was done after the native preprocessed images being warped into standard space.

### Multimodal images normalization

Similar spatial registration steps were done for both native diffusion images and native function images using the Advanced Normalization Tools (ANTs).^4^ First, the b0 volume or the first function volume were skull-stripped and non-linearly warped to the individual anatomical volume with high resolution. The non-linear co-registration here was thought to better control the EPI distortion in both diffusion and function images. Second, the native anatomical volume was non-linearly warped into to the University of North Carolina (UNC) brain standard space for 2-year-olds (Shi et al., 2011). All the spatial transform files were combined into one step warping in order to avoid multiple interpolations. Although the subjects in current study were aged across 6 years, within which the brain possess a fast and complex growth, we chose a single 2-year-old template as the appropriate target space here considering two aspects: (1) newborn brain grows logarithmically with the major growth before and relatively gentle growth after about 2-year-old; (2) the 2-year-old brain is almost adultlike and only two newborns were less than 1-year-old.

### Network construction

Totally 90 regions covering the brain from the Automated Anatomical Labeling (AAL) atlas (Shi et al., 2011) were labeled and used to build both the structural and functional connectivity networks. Structural connectivity was generated with DSI studio^5^ (Yeh et al., 2013a,b) with the deterministic fiber assignment by continuous tracking (FACT) algorithm. The fiber tracking terminated if one of these conditions were met: track length shorter than 10 or longer than 200 mm; FA threshold lower than 0.1; turning angle higher than 45°. The normalized number of fiber streamlines between paired regions by the total number of streamlines, as well as the mean DTI metrics of voxels that the tract passing, were computed, yielding five networks with different structural measures. Functional connectivity was quantified with the Fisher-Z transformed Pearson’s correlation of the preprocessed bold signals from paired regions. In order to obtain the spatial location relationship between regions, Euclidean distance between each paired regions were calculated in 2-year-old standard space.

### Brain structural-functional coupling

Regional connectivity profiles were extracted from each column of a subject’s functional connectivity matrix and five structural connectivity matrixes. Multiple linear regression analysis was performed on these connectivity profiles, predicting the regional functional connectivity profile from the structural connectivity profiles. All variables were normalized to be zero mean and one standard deviation before regression modeling. Structure-function coupling score was then measured as the adjusted R-square score of the model fitting for each region (Wang et al., 2018; Baum et al., 2020). Furthermore, we grouped the whole brain connections into short connections and long connections by the Euclidean distance threshold of about 66 mm, which was the mean length between regions in 2-year-old standard space (Shi et al., 2011). Similarly, two more structure-function coupling scores for short connections and for long connections were generated for each region.

## Statistical analysis

### Analysis of demographic data

The continuous data of the patients with IE and healthy controls were compared by two-sample *t*-test. The categorical data of the two groups were compared by Fisher exact test. A statistical tool SPSS22.0 (SPSS Inc., Chicago, IL, United States)^6^ was applied for the analysis mentioned above. *P* < 0.05 was considered statistically significant between the two groups.

### Analysis of coupling profile of each node

Two-sample *t*-tests were performed to compare the node wised coupling score of IE patients with that of healthy controls at different network scales (i.e., whole brain connectome, short-distance connectome, and long-distance connectome). In addition, the age association of the coupling score for different connectomes was assessed through Pearson correlation analysis in both groups, respectively. To identify the distinct developmental trends of coupling metrics in IE patients, we evaluated the interaction effect between group (IE group vs. healthy group) and age in terms of coupling score, which was performed by a linear mixed effects model. More specifically, in the linear mixed effects model, coupling score was taken for the dependent variable while age, gender, group, and their interaction items were regarded as fixed effects, with random slope and random intercept for groups as random effects. A *P*-value of < 0.05 was considered for establishing the statistical significance, with correction for multiple comparation by the algorithm of false discovery rate (FDR).

### Correlation analysis of coupling score with clinical variable

For patients with IE, we evaluated the correlation of coupling score for different connectomes of each node with strabismus degree by Pearson correlation analysis. An FDR-corrected *P*-value of < 0.05 was considered statistically significant. We also examined the age-related regulatory effect on the association between coupling score and strabismus degree, applying hierarchical regression analysis in each region. Specifically, at first, one initial model was established with an independent variable of strabismus degree and a dependent variable of coupling score. Then the other model was built on the basis of the first one, adding age as a covariate. Finally, the changes between two models were examined to assess the regulatory effect of age. Given that these analyses were exploratory in nature, in this step, an uncorrected *P*-value of 0.05 was set as the significance threshold, without regard to the correction for multiple comparison.

## Results

### Demographic and clinical data

The demographic and clinical data of the patients with IE and healthy controls were listed in Table 1. No significant difference was found between the two groups in age or gender (both *P* > 0.05).

**TABLE 1:** Demographical and clinical features of the subjects.

	IEs (*n* = 18)	HCs (*n* = 20)	*P*-value
Age (years)	3.27 ± 2.00	3.41 ± 1.58	0.812^a^
Sex, male/female	10/8	11/9	>0.99^b^
Onset age (months)	4.17 ± 1.72	N/A	N/A
Duration (months)	35.00 ± 23.53	N/A	N/A
Strabismus degree (PD)	54.44 ± 18.93	N/A	N/A

IEs, infantile esotropia patients; HCs, healthy controls; N/A, not applicable; PD, prism degree.

^a^Two-sample two-tailed *t*-test.

^b^Fisher exact test.

### Between-group difference in SC-FC coupling profile

**FIGURE 1:**
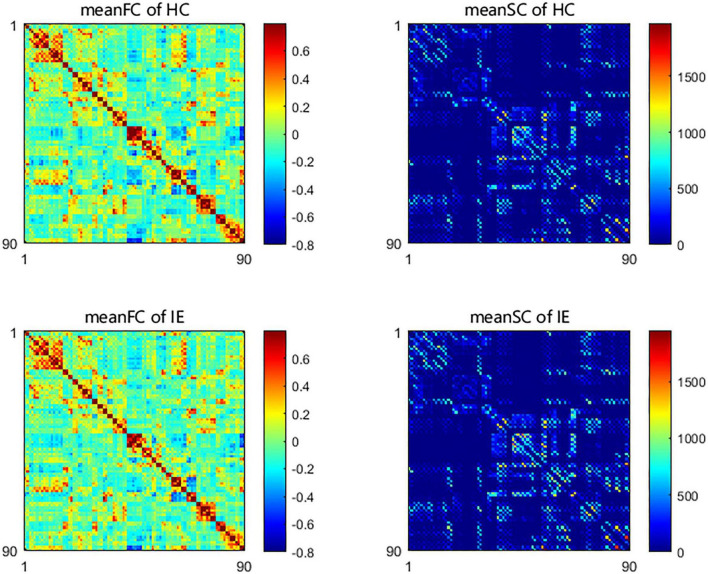
Group-averaged network connectivity matrices based on Automated Anatomical Labeling (AAL) atlas in patients with infantile esotropia and healthy participants. The functional connectivity matrices were constructed with link weights of correlation between time series from pairs of brain areas. The structural connectivity matrices were established with edge weights of fractional streamline count between paired brain regions. SC, structural connectivity; FC, functional connectivity; HC, healthy control; IE, infantile esotropia.

**FIGURE 2:**
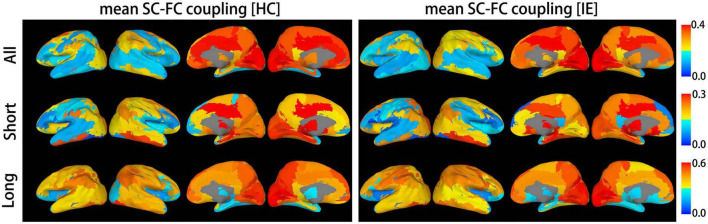
Mean structural-functional connectivity coupling score of each brain subregion. The graphic presents mean coupling score for whole brain connections, long-distance connections and short-distance connections of each brain subregion in patients with infantile esotropia and healthy subjects. SC, structural connectivity; FC, functional connectivity; HC, healthy control; IE, infantile esotropia.

**FIGURE 3:**
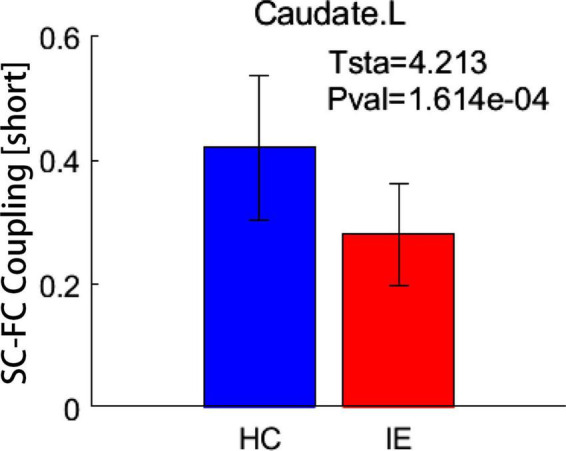
Between-group difference in coupling metrics. Patients with infantile esotropia exhibited attenuated coupling score for short connections in left caudate nucleus relative to healthy subjects (*P* < 0.05, FDR corrected). The whisker represents the standard deviation of the mean. SC, structural connectivity; FC, functional connectivity; HC, healthy control; IE, infantile esotropia; L, left.

Both the structural and functional connectivity matrices of IE patients and healthy subjects were shown in Figure 1. Additional structural connectivity matrixes derived from FA, MD, AD, and RD of streamlines between brain subregions were presented in Supplementary Figure 1. SC-FC coupling scores for whole brain connections, short connections and long connections of each brain subregion were obtained in patients with IE as well as healthy controls, which were shown in Figure 2. For short connectome, the coupling score of left caudate nucleus (CAU) decreased in patients with IE relative to healthy subjects (*P* < 0.05, FDR-corrected) (Figure 3). However, none of brain subregions showed significant between-group difference that survived multiple comparation correction, in terms of coupling metrics for global or long connectome (Supplementary Figure 2).

### Developmental trends of coupling score in IE patients and healthy controls

**FIGURE 4:**
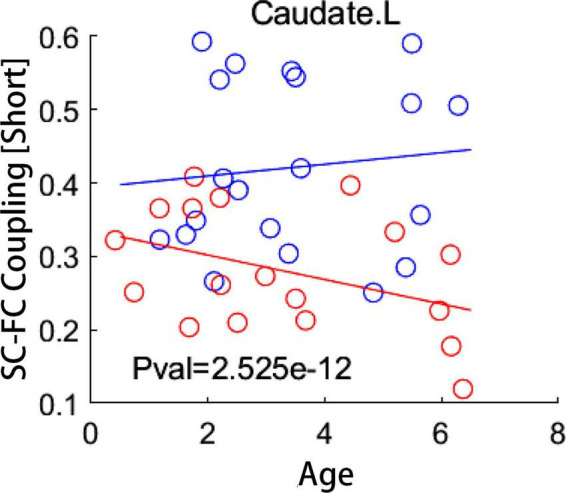
Group-age interaction of coupling score examined by a linear mixed effect model. Compared to healthy subjects, patients with infantile esotropia showed distinct developmental trajectory of local coupling measures in left caudate nucleus (FDR-corrected *P* < 0.05). Blue circles indicate healthy controls while red circles represent patients with infantile esotropia. SC, structural connectivity; FC, functional connectivity; L, left.

Age-related changes of SC-FC coupling score of numerous brain regions were found in IE patients and healthy controls, respectively. Details of these results were presented in Supplementary Figure 3. To determine which brain regions showed distinct development trajectory of SC-FC coupling in IE patients compared to healthy controls, we applied a linear mixed effects model approach to examine the interactive effect between the group and age in terms of coupling score. None but the left CAU exhibited significant group-age interaction in SC-FC coupling for short connections following correction for multiple comparation (FDR-corrected *P* < 0.05) (Figure 4). Detailed results of interactive effect analysis before multiple comparation correction were also rendered in Supplementary Figure 4.

### Correlation of coupling score with degree of strabismus in IE patients

**FIGURE 5:**
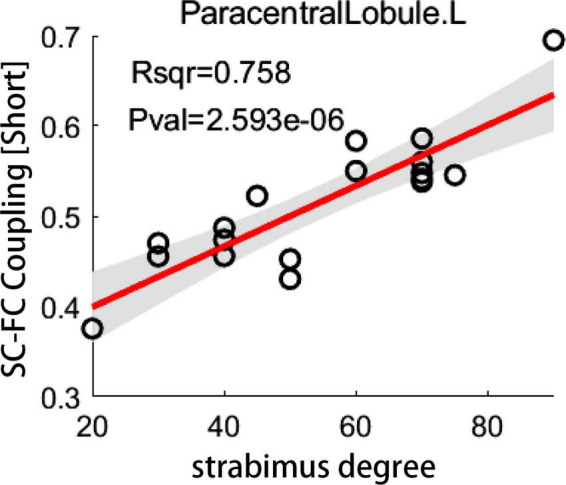
The correlation between coupling score and strabismus degree. Scatter plot depicts a significantly positive correlation between coupling score for short connections in left paracentral lobule and strabismus degree in patients with infantile esotropia (*P* < 0.05, corrected by FDR). SC, structural connectivity; FC, functional connectivity; L, left.

For short connectome, the coupling score of left paracentral lobule (PCL) positively correlated with the degree of esodeviation (*R*^2^ = 0.758; *P* < 0.05, corrected by FDR) (Figure 5). For global or long connectome, no significant correlation between coupling score and strabismus degree survived the FDR correction (Supplementary Figure 5).

**FIGURE 6:**
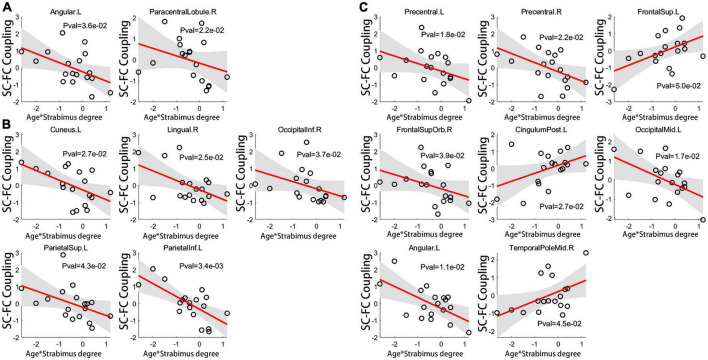
The age-related regulatory effect on correlation of coupling score for global connections **(A)**, long connections **(B)** and short connections **(C)** with strabismus degree. For brain regions implicated in visual or sensorimotor process, the relationship between coupling score and strabismus degree became looser with age. On the contrary, for cognition-related brain regions, an age-dependent strengthened association was evident between coupling score and strabismus degree (unadjusted *P* < 0.05). Brain regions and their abbreviations are detailed in Supplementary Table 1. SC, structural connectivity; FC, functional connectivity; L, left; R, right.

Interestingly, there was a significant regulatory effect of age on the correlation between coupling score and strabismus degree in patients with IE. Specifically, age-dependent declined correlation between coupling score and strabismus degree was mainly observed in visual or sensorimotor brain subregions, such as left angular gyrus, right PCL, bilateral precentral gyri, right orbital part of superior frontal gyrus, left middle occipital gyrus, left cuneus, right lingual gyrus, right inferior occipital gyrus, left superior parietal gyrus, and left inferior parietal lobule. In contrast, age-related increased correlation between coupling score and strabismus degree was evident in core components of the default mode network, including left superior frontal gyrus, left posterior cingulate gyrus and right temporal pole of middle temporal gyrus (Figure 6). Details of brain regions and their abbreviations were documented in Supplementary Table 1.

## Discussion

This is the first study exploring the potential neural basis of pathogenesis of IE from the point of brain structural and functional measures interaction, through a combined DTI and rsfMRI analysis. Our main findings were as follows: (1) patients with IE exhibited aberrant SC-FC coupling in CAU compared to healthy subjects; (2) A decoupled tendency with age in structure-function relationship of CAU was initiated in newborn period and persisted throughout early childhood in IE patients; (3) For SC-FC coupling profile, it was short connectome, but not the long connectome, that was prone to be disturbed under the influence of IE; (4) The coupling measures of the sensorimotor region (i.e., PCL) showed significantly positive correlation with the strabismus degree; (5) age-related regulatory effect on the association between coupling score and strabismus degree was discriminative in brain areas related to visual or sensorimotor processing and those responsible for cognitive tasks.

It is considered that structural and functional network interact with one another. More specifically, the SC acts as the anatomical infrastructure and imposes constraints for the FC, while FC exerts effect on shaping of the SC through a plasticity mechanism (Honey et al., 2009; Guerra-Carrillo et al., 2014). The attenuated SC-FC coupling of the CAU arises from the weakened association between SC and FC. In fact, CAU plays a central role in visual processing. On the one hand, it has been reported to be involved in the static and dynamic visual information processing (Gombkötõ et al., 2013), equilibrating visual and reward information (Doi et al., 2020) and visual category learning (Nomura and Reber, 2008). Moreover, CAU is capable of receiving the visual content information and visuospatial information, and thus is an indispensable component implicated in both ventral and dorsal visual pathway (Yamamoto et al., 2012). Although a weaker structure-function coupling may contribute to the more flexible function, it may make some areas pertaining to the visual pathway (e.g., the CAU) vulnerable in the presence of heterogeneous binocular inputs, which accounts for the observed abnormality in stereopsis in IE patients (Zarkali et al., 2021). Impairment in voxel-mirrored homotopic connectivity or amplitude of low-frequency fluctuation in CAU was also proved in patients with strabismus (Yin et al., 2021; Zhang et al., 2021). Of note, it was left ACU, but not the bilateral CAU, that showed anomalous coupling metrics in IE patients. The cerebral-lateralization theory may be reasonable for this observation, but the underlying mechanism needs to be further explored in future.

On the other hand, direct pathway projections are existent between the CAU and substantia nigra pars reticulata, through an open loop pathway, the latter projects to the visual reflex center, that is, the superior colliculus, which is connected with lateral geniculate nucleus *via* the brachium of superior colliculus and associates reticular structure of brainstem through afferent fibers, with responsibility for the eye movement regulation and eye position control (Seger, 2013). As an important part of the visual corticostriatal loop, CAU is also closely linked with visual temporal cortex that encodes visual motion (Choi et al., 2012; Rolls et al., 2022). Overall, convergent evidences suggest that CAU not only involves in the visual corticostriatal loop (Lawrence et al., 1998), but also is implicated in subcortical visual motion circuit (Brodsky, 2019). Thus, we speculated that alterations in SC-FC coupling of CAU could interfere the visual corticostriatal loop responsible for visual processing and eye movement planning as well as the visual subcortical circuit subserving nasalward optokinesis, resulting in visuospatial impairment and nasalward deviation of eyes in IE patients, which lends neuroimaging support to the recent hypothesis that changed plasticity of subcortical visual pathway during the newborn period in IE patients induce monocular asymmetric optokinetic response to the horizontal stimulus and thus drive the nasalward rotation of both eyes (Brodsky, 2019).

An open question is that when the SC-FC coupling deficits appear in children with IE during the process of neurodevelopment. In this study, we found that the SC-FC coupling changes with age during typical development, with distinct trajectories in different brain regions, as similar to previous studies (Collin and van den Heuvel, 2013). Interestingly, the developmental trends of SC-FC coupling across age were also demonstrated in patients with IE in several brain regions, among which the CAU exhibited entirely different trajectory in coupling metrics compared to that of the healthy counterparts. More importantly, our findings also suggested that the disturbance of SC-FC coupling evolution was initiated in their early postnatal period, which enhanced the understanding for the developmental pattern of network property in patients with IE and provided evidence to explain the universal phenomenon that most IE patients present inward deviation of eyes within the first 6 months of life.

For IE patients, enhanced SC-FC coupling in PCL significantly correlated with increased strabismus degree. The heightened SC-FC coupling in PCL in patients with IE may indicate that functional connections through the PCL are more prone to rely on the intrinsic structural connections, resulting in less dynamic brain function (Dai et al., 2019). The PCL, known as an important part of the sensorimotor network, plays a central role in the sensorimotor integration, which contributes to the visuomotor coordination (Di Ciò et al., 2020). Increased functional dependence over structural connectivity in PCL as the strabismus degree increasing indicates the more serious the esotropia is, the less flexible is the brain function dynamics in PCL, which may contribute to the observed abnormalities in hand-eye coordination in IE children. Anomalous cortical thickness or voxel-wise degree centrality in PCL was also reported in patients with strabismus (Wu et al., 2020; Yin et al., 2021). Interestingly, for visual-related or sensorimotor cortical areas, the correlation between the coupling score and strabismus degree had a downtrend with the increase of age, indicating that the younger the IE patients are, the greater influence the strabismus degree exerts on SC-FC coupling of the visual or sensorimotor regions. Given the fact of early onset of IE, corrective surgery is recommended to be implemented as early as possible to relieve the disturbance of coupling arising from esotropia. On the other hand, for cortical regions pertaining to cognition (i.e., such pivotal hubs of default mode network as superior frontal gyrus, posterior cingulate gyrus, and middle temporal gyrus), the association between the coupling metrics and strabismus degree displayed a growing tendency with age, which means the older the patients are, the more serious is the cognitive impairment related to esotropia, suggesting that misalignment of eyes may bring continuous undesirable effect on the cognition development (Zhen et al., 2018).

Previous studies have demonstrated that SC-FC coupling profile is influenced by the distances between brain areas which interconnect with each other (Collin et al., 2017; Zhang et al., 2019). Likewise, our observations showed that the SC-FC coupling of patients with IE was mainly affected in short connections, but rather in long connections, across the whole brain subregions. One reasonable explanation is that, the local connectome responsible for visual processing is more vulnerable to unequal visual input from both eyes. As a congenital fusion-deficient strabismus, IE exhibits evident deficits in stereovision and eye movement, which may be attributed to the disturbed SC-FC coupling in local regions with short connections linked to visual pathway.

Several limitations should be noted when interpreting the results. First, a relatively small number of subjects was enrolled in the present study. Even so, this study has been the largest one in sample size among the advanced neuroimaging (i.e., fMRI, DTI, and so forth) studies for patients with IE up to now and, for the first time, offers insight into the interaction between structural and functional networks in IE patients. Second, the age-related regulatory effect analysis on the coupling-degree association was exploratory in nature. In this context, correction for multiple comparation was not employed in these analyses, which may inevitably bring about some false positive entries, thus some cautions are essential when illustrating these observations. Additional topological properties of the network would be explored in future works through a graph theoretical method, since some of which play an important role in the global information communication and has been reported to have associations with SC-FC coupling metrics in both healthy and disease states (Collin et al., 2014; Kim et al., 2019; Cao et al., 2020).

## Conclusion

Patients with IE exhibited decoupled local SC-FC association in CAU, which was initiated from the early postnatal period and aggravated with age. Altered SC-FC coupling in CAU in IE patients could interfere the normal visual corticostriatal loop and subcortical optokinetic pathway, causing monocular nasal-temporal optokinetic asymmetry and driving the eyes into nasalward positions, which may be the underlying pathogenesis of IE. To the best of our knowledge, the present study offered first evidence of disrupted coupling profile of CAU in IE, which could enhance our understanding for the neurobiological process of IE. Increased esotropia degree was associated with decreased functional dynamics of brain’s sensorimotor region, which could bring about the anomaly in visuomotor coordination. The influence of esotropia on SC-FC coupling is prominent for brain regions related to visual or sensorimotor processing for children in their very early years and is accumulative for cognition-related brain regions across childhood. Future studies with larger sample size and additional network topological properties analyses are needed to verify these findings.

## Data availability statement

The raw data supporting the conclusions of this article will be made available by the authors, without undue reservation.

## Ethics statement

The studies involving human participants were reviewed and approved by Medical Research Ethic Committee of Beijing Children’s Hospital. Written informed consent to participate in this study was provided by the participants’ legal guardian/next of kin.

## Author contributions

YP, NL, and DH designed the study. JG was in charge of acquiring imaging data and drafting the manuscript. WL, LH, YL, and HK contributed to the recruiting participants and collecting clinical data. YC was responsible for data analysis. All authors approved the final manuscript.

## Footnotes

1
https://fsl.fmrib.ox.ac.uk/fsl/fslwiki/

2
https://surfer.nmr.mgh.harvard.edu/

3
https://afni.nimh.nih.gov/

4
http://stnava.github.io/ANTs/

5
https://dsi-studio.labsolver.org/

6
https://www.ibm.com/products/spss-statistics
