# Supplementary material for: Local structural-functional connectivity decoupling of caudate nucleus in infantile esotropia
Source: Front Neurosci. 2022 Dec 22;16:1098735. doi: 10.3389/fnins.2022.1098735 (PMC9815444; doi:10.3389/fnins.2022.1098735)
Supplement: Supplementary file 1 [file Data_Sheet_1.PDF]

Supplementary Material

Table S1. Regions of interest labeled by AAL brain atlas

Labels	Regions	Regions	Abbreviations
1	Precentral_L	Precentral gyrus	PreCG.L
2	Precentral_R	Precentral gyrus	PreCG.R
3	Frontal_Sup_L	Superior frontal gyrus, dorsolateral	SFGdor.L
4	Frontal_Sup_R	Superior frontal gyrus, dorsolateral	SFGdor.R
5	Frontal_Sup_Orb_L	Superior frontal gyrus, orbital part	ORBsup.L
6	Frontal_Sup_Orb_R	Superior frontal gyrus, orbital part	ORBsup.R
7	Frontal_Mid_L	Middle frontal gyrus	MFG.L
8	Frontal_Mid_R	Middle frontal gyrus	MFG.R
9	Frontal_Mid_Orb_L	Middle frontal gyrus, orbital part	ORBmid.L
10	Frontal_Mid_Orb_R	Middle frontal gyrus, orbital part	ORBmid.R
11	Frontal_Inf_Oper_L	Inferior frontal gyrus, opercular part	IFGoperc.L
12	Frontal_Inf_Oper_R	Inferior frontal gyrus, opercular part	IFGoperc.R
13	Frontal_Inf_Tri_L	Inferior frontal gyrus, triangular part	IFGtriang.L
14	Frontal_Inf_Tri_R	Inferior frontal gyrus, triangular part	IFGtriang.R
15	Frontal_Inf_Orb_L	Inferior frontal gyrus, orbital part	ORBinf.L
16	Frontal_Inf_Orb_R	Inferior frontal gyrus, orbital part	ORBinf.R
17	Rolandic_Oper_L	Rolandic operculum	ROL.L
18	Rolandic_Oper_R	Rolandic operculum	ROL.R
19	Supp_Motor_Area_L	Supplementary motor area	SMA.L
20	Supp_Motor_Area_R	Supplementary motor area	SMA.R
21	Olfactory_L	Olfactory cortex	OLF.L
22	Olfactory_R	Olfactory cortex	OLF.R
23	Frontal_Sup_Medial_L	Superior frontal gyrus, medial	SFGmed.L
24	Frontal_Sup_Medial_R	Superior frontal gyrus, medial	SFGmed.R
25	Frontal_Mid_Orb_L	Superior frontal gyrus, medial orbital	ORBsupmed.L
26	Frontal_Mid_Orb_R	Superior frontal gyrus, medial orbital	ORBsupmed.R
27	Rectus_L	Gyrus rectus	REC.L
28	Rectus_R	Gyrus rectus	REC.R
29	Insula_L	Insula	INS.L
30	Insula_R	Insula	INS.R
31	Cingulum_Ant_L	Anterior cingulate and paracingulate gyri	ACG.L
32	Cingulum_Ant_R	Anterior cingulate and paracingulate gyri	ACG.R
33	Cingulum_Mid_L	Median cingulate and paracingulate gyri	DCG.L
34	Cingulum_Mid_R	Median cingulate and paracingulate gyri	DCG.R
35	Cingulum_Post_L	Posterior cingulate gyrus	PCG.L
36	Cingulum_Post_R	Posterior cingulate gyrus	PCG.R
37	Hippocampus_L	Hippocampus	HIP.L
38	Hippocampus_R	Hippocampus	HIP.R
39	ParaHippocampal_L	Parahippocampal gyrus	PHG.L
40	ParaHippocampal_R	Parahippocampal gyrus	PHG.R
41	Amygdala_L	Amygdala	AMYG.L
42	Amygdala_R	Amygdala	AMYG.R
43	Calcarine_L	Calcarine fissure and surrounding cortex	CAL.L
44	Calcarine_R	Calcarine fissure and surrounding cortex	CAL.R
45	Cuneus_L	Cuneus	CUN.L
46	Cuneus_R	Cuneus	CUN.R
47	Lingual_L	Lingual gyrus	LING.L
48	Lingual_R	Lingual gyrus	LING.R
49	Occipital_Sup_L	Superior occipital gyrus	SOG.L
50	Occipital_Sup_R	Superior occipital gyrus	SOG.R
51	Occipital_Mid_L	Middle occipital gyrus	MOG.L
52	Occipital_Mid_R	Middle occipital gyrus	MOG.R
53	Occipital_Inf_L	Inferior occipital gyrus	IOG.L

54	Occipital_Inf_R	Inferior occipital gyrus	IOG.R
55	Fusiform_L	Fusiform gyrus	FFG.L
56	Fusiform_R	Fusiform gyrus	FFG.R
57	Postcentral_L	Postcentral gyrus	PoCG.L
58	Postcentral_R	Postcentral gyrus	PoCG.R
59	Parietal_Sup_L	Superior parietal gyrus	SPG.L
60	Parietal_Sup_R	Superior parietal gyrus	SPG.R
61	Parietal_Inf_L	Inferior parietal, but supramarginal and angular gyri	IPL.L
62	Parietal_Inf_R	Inferior parietal, but supramarginal and angular gyri	IPL.R
63	SupraMarginal_L	Supramarginal gyrus	SMG.L
64	SupraMarginal_R	Supramarginal gyrus	SMG.R
65	Angular_L	Angular gyrus	ANG.L
66	Angular_R	Angular gyrus	ANG.R
67	Precuneus_L	Precuneus	PCUN.L
68	Precuneus_R	Precuneus	PCUN.R
69	Paracentral_Lobule_L	Paracentral lobule	PCL.L
70	Paracentral_Lobule_R	Paracentral lobule	PCL.R
71	Caudate_L	Caudate nucleus	CAU.L
72	Caudate_R	Caudate nucleus	CAU.R
73	Putamen_L	Lenticular nucleus, putamen	PUT.L
74	Putamen_R	Lenticular nucleus, putamen	PUT.R
75	Pallidum_L	Lenticular nucleus, pallidum	PAL.L
76	Pallidum_R	Lenticular nucleus, pallidum	PAL.R
77	Thalamus_L	Thalamus	THA.L
78	Thalamus_R	Thalamus	THA.R
79	Heschl_L	Heschl gyrus	HES.L
80	Heschl_R	Heschl gyrus	HES.R
81	Temporal_Sup_L	Superior temporal gyrus	STG.L
82	Temporal_Sup_R	Superior temporal gyrus	STG.R
83	Temporal_Pole_Sup_L	Temporal pole: superior temporal gyrus	TPOsup.L
84	Temporal_Pole_Sup_R	Temporal pole: superior temporal gyrus	TPOsup.R
85	Temporal_Mid_L	Middle temporal gyrus	MTG.L
86	Temporal_Mid_R	Middle temporal gyrus	MTG.R
87	Temporal_Pole_Mid_L	Temporal pole: middle temporal gyrus	TPOmid.L
88	Temporal_Pole_Mid_R	Temporal pole: middle temporal gyrus	TPOmid.R
89	Temporal_Inf_L	Inferior temporal gyrus	ITG.L
90	Temporal_Inf_R	Inferior temporal gyrus	ITG.R

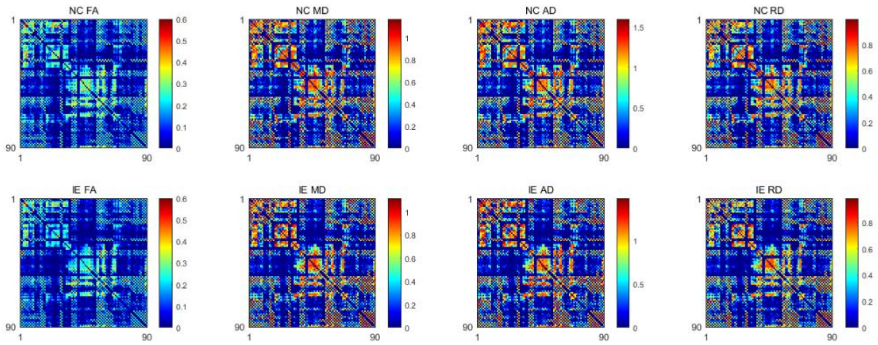

Figure S1. Structural connectivity matrixes based on different streamline properties. The graphic shows mean structural connectivity matrixes derived from different metrics of fiber bundles between paired brain regions in patients with infantile esotropia and healthy subjects, which comprise fractional anisotropy matrixes, mean diffusivity matrixes, axial diffusivity matrixes and radial diffusivity matrixes. NC, normal control; IE, infantile esotropia; FA, fractional anisotropy; MD, mean diffusivity; AD, axial diffusivity; RD, radial diffusivity.

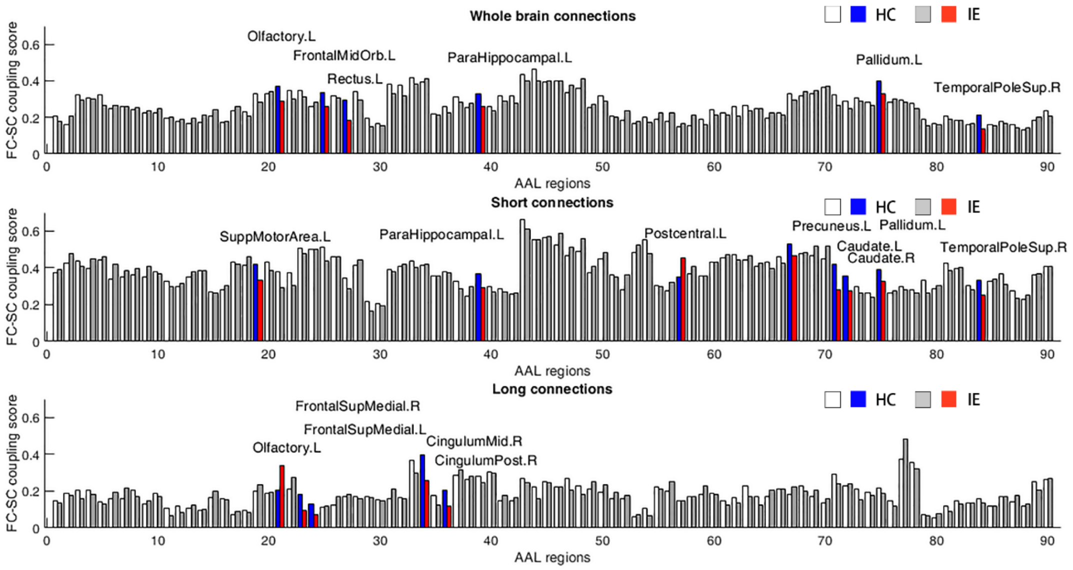

Figure S2. The difference between patients with infantile esotropia and healthy subjects in coupling metrics. For whole brain connections, decreased SC-FC coupling score of left olfactory cortex, left medial orbital part of superior frontal gyrus, left gyrus rectus, left parahippocampal gyrus, left pallidum and right temporal pole of superior temporal gyrus was observed in patients with IE compared to healthy subjects. For short connections, the SC-FC decoupling was evident in IE patients in such areas as left supplementary motor area, left parahippocampal gyrus, left precuneus, bilateral caudate nuclei, left pallidum and right temporal pole of superior temporal gyrus, in contrast to the increased coupling score in left postcentral gyrus. For long connections, a decline in coupling score was found in IE patients in several brain regions consisting of bilateral medial part of superior frontal gyri, right median cingulate gyrus and right posterior cingulate, in contrast to the increase in coupling score of left olfactory cortex. (unadjusted $P<0.05$). SC, structural connectivity; FC, functional connectivity; HC, healthy control; IE, infantile esotropia; L, left; R, right.

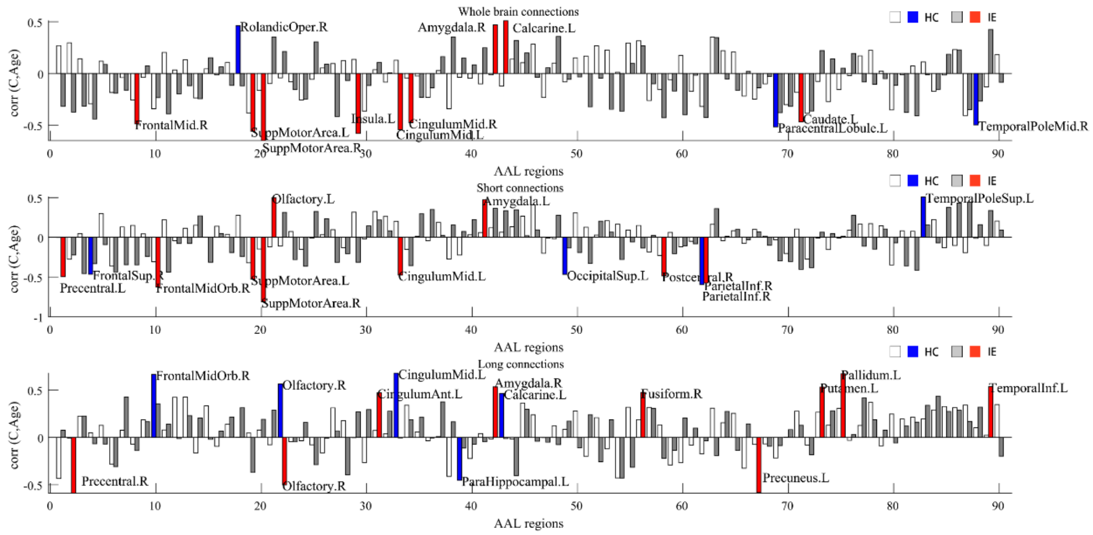

Figure S3. The correlation of coupling score with age in patients with infantile esotropia and healthy subjects. Age-related changes of SC-FC coupling score of each brain region were evaluated in IE patients and healthy controls, respectively. Specifically, for global connectome, increased coupling score with age was observed in IE patients in right amygdala (AMYG) and left calcarine fissure, in contrast to the decrease in many regions which consists of right middle frontal gyrus, bilateral supplementary motor areas, left insula, bilateral median cingulate gyri (DCG) and left caudate nucleus; In healthy subjects, age-related increased coupling score was found in right Rolandic operculum in contrast to the decline in left paracentral lobule and right temporal pole of middle temporal gyrus. For short connectome, an increase of coupling score with age was found in IE patients in left olfactory cortex and left AMYG in contrast to the decline in several brain regions including left precentral gyrus, right orbital part of middle frontal gyrus, bilateral supplementary motor areas, left DCG, right postcentral gyrus and right inferior parietal lobule; In heathy subjects, an age-related increase in coupling score was evident in left temporal pole of superior temporal gyrus in contrast to the decrease in right superior frontal gyrus, left superior occipital gyrus and right inferior parietal lobule. For long connectome, increased coupling score with age was demonstrated in IE patients in several brain areas, such as left anterior cingulate gyrus, right AMYG, right fusiform gyrus, left putamen, left pallidum and left inferior temporal gyrus, in contrast to the decline in right precentral gyrus, right olfactory cortex and left precuneus; In healthy subjects, an age-related increase in coupling score was shown in right orbital part of middle frontal gyrus, right olfactory cortex, left DCG and left calcarine fissure, in contrast to the decrease in left parahippocampal gyrus.
HC, healthy control; IE, infantile esotropia; L, left; R, right; C, coupling.

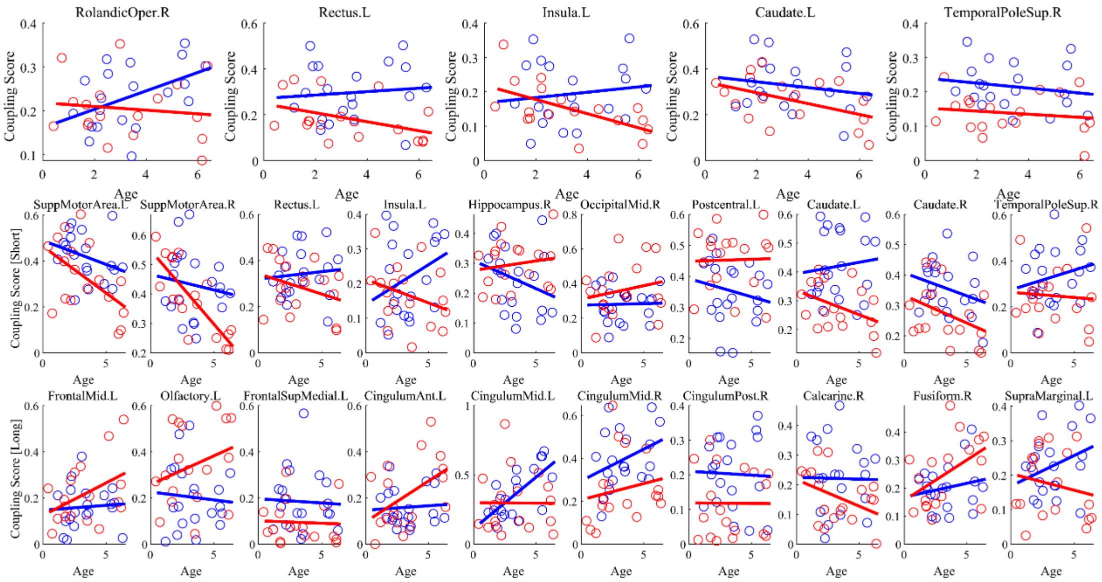

Figure S4. Group-age interaction for coupling measures. The group-age interaction of coupling score was observed in some brain areas (i.e., right Rolandic operculum, left gyrus rectus, left insula, left caudate nucleus, right temporal pole of superior temporal gyrus) for whole brain connections, in numerous regions (i.e., bilateral supplementary motor areas, left gyrus rectus, left insula, right hippocampus, right middle occipital gyrus, left postcentral gyrus, bilateral caudate nuclei and right temporal pole of superior temporal gyrus) for short connections, in several brain regions (i.e., left middle frontal gyrus, left olfactory, left medial part of superior frontal gyrus, left anterior cingulate gyrus, bilateral median cingulate gyri, right posterior cingulate gyrus, right calcarine fissure, right fusiform gyrus and left supramarginal gyrus) for long connections. Red: infantile esotropia; Blue: healthy control. L, left; R, right.

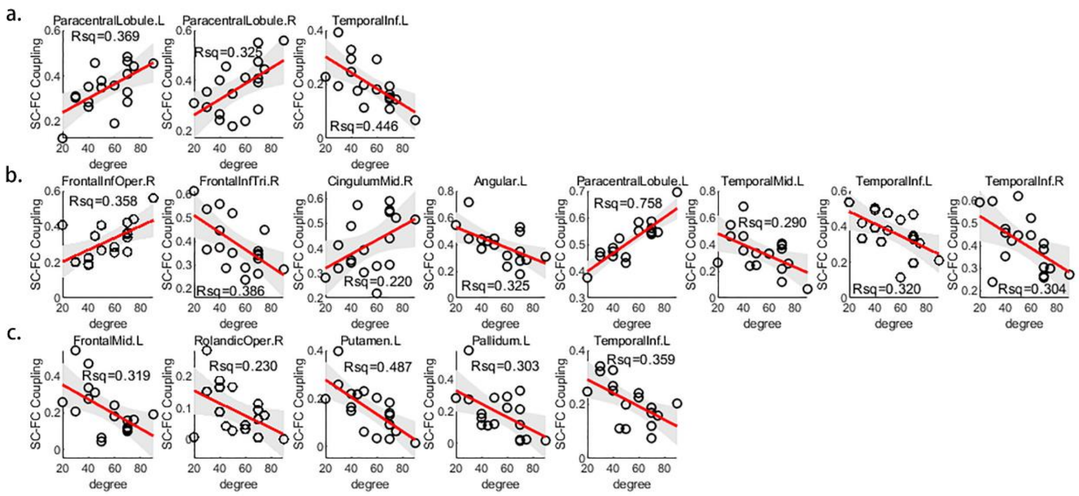

Figure S5. Correlation between coupling score and strabismus degree. Before multiple comparison correction, there was a significant correlation of strabismus degree with global SC-FC coupling score in bilateral paracentral lobule and left inferior temporal gyrus (a), with short-distance SC-FC coupling score in opercular and triangular part of right inferior frontal gyrus, right median cingulate gyrus, left angular gyrus, left paracentral lobule, left middle temporal gyrus and bilateral inferior temporal gyri (b), with long-distance SC-FC coupling score in left middle frontal gyrus, right Rolandic operculum, left putamen, left pallidum and left inferior temporal gyrus (c).
